# Supplementary material for: Are carbon emissions trading and green financial instruments synergistic? -Comprehensive quantitative research based on content analysis
Source: PLoS One. 2024 Mar 7;19(3):e0298601. doi: 10.1371/journal.pone.0298601 (PMC10919601; doi:10.1371/journal.pone.0298601)
Supplement: S1 Data — (ZIP) [file pone.0298601.s001.zip › Data/地级市Z_F工作报告.zip!/░▓╗╒/═¡┴Ω╩╨/└┤╫╘╣1⁄2╓┌║┼í╛┬φ┐╦╩2╛▌═°í┐_2017.docx]

各位代表：

　　现在，我代表市人民政府，向大会报告工作，请予审议，并请市政协委员和其他列席人员提出意见和建议。

　　一、2016年工作回顾

　　过去的一年，是一江两岸新铜陵扬帆起航之年。全市人民在省委、省政府和中共铜陵市委的坚强领导下，深入贯彻党的十八大和十八届历次全会精神，全面落实习近平总书记系列重要讲话特别是视察安徽重要讲话精神，坚持以新发展理念引领发展行动，适应把握引领经济发展新常态，坚持稳中求进工作总基调，主动应对经济持续下行压力，组织实施“生态建设提升年”、“规划引领突破年”、“精准扶贫和棚改攻坚年”活动，统筹推进各项工作，负重奋进、砥砺前行，圆满完成市十五届人大六次会议确定的主要目标任务。经济运行稳中有进，主要指标好于预期，社会大局和谐稳定，顺利实现“十三五”良好开局。

　　预计，全年实现地区生产总值960亿元，增长9%，增幅居全省第6位。财政收入153.8亿元，增长5%，其中地方收入增长8.4%。固定资产投资1196亿元，增长12.5%，增幅居全省第5位。社会消费品零售总额304.4亿元，增长11.8%。城镇居民收入30460元，农村居民人均可支配收入12200元，分别增长8.8%、9.2%。节能减排实现年度控制目标，万元GDP能耗降幅居全省前列。

　　这一年，我们顺利完成行政区划重大调整。枞阳县、铜官区、义安区区划调整平稳有序，高效实施。江南江北发展规划、思想观念等加速融合，工作标准、民生待遇等加速接轨。两岸协调发展全面布局，全省新建里程最长的国道347等重大项目陆续开工，枞阳人民期盼多年的梦想正在化为现实。

　　这一年，我们推动转型发展取得重大成效。产业加快向中高端迈进，战略性新兴产业产值占规上工业比重由28.8%提高到30.6%，居全省第2位；铜材加工突破175万吨，超过电解铜产量43万吨；铜产业朝全链条、高价值方向延伸，新能源汽车从部件生产向整车制造、智能制造迈进，电子信息产业由元器件、印制电路板向手机等终端产品拓展。服务业增加值占GDP比重35.4%，提高2.3个百分点。国家节能减排示范市绩效连续两年考核优秀，资源型城市转型是唯一连续三年获得全国优秀的城市，打造循环经济升级版做法在全国推广。

　　这一年，我们争创国家各类试点示范取得重大成果。狮子山国家高新区即将获得国务院批准。铜官区成为国家服务业综合改革试点区。我市成为全国社会工作服务综合示范市、中央财政支持开展居家和社区养老服务改革试点地区。西湖国家城市湿地公园命名在即。国家森林城市创建基本达标。大通镇成为首批中国特色小镇。枞阳县获批创建国家全域旅游示范区。群心村成为我市第二个全国美丽宜居示范村庄。全国文明城市建设年度测评成绩名列全省前茅。全国双拥模范城实现“四连冠”。国家公共文化服务体系示范区创建受到文化部好评。智慧城市建设成效显著，城市社区社会管理服务信息化实现全覆盖。

　　这一年，我们奋力夺取抗洪救灾重大胜利。面对历史罕见的降雨强度、河湖水位和险情灾情，我们紧急动员，周密部署，及时转移安置群众5万多人；全市上下万众一心，奋力抢险，实现了人员不伤亡、水库不垮坝、万亩圩口不决堤的目标。灾后迅速启动恢复重建，744户倒房户11月底前全部“回家”，3500多处水毁设施完成修复，受灾群众生产生活安排有序有力有效。

　　一年来，我们主要做了以下工作：

　　第一，围绕提质增效，扎实推进供给侧改革。坚持优化存量、引导增量、主动减量，全面实施供给侧结构性改革总体方案和配套措施，着力推进“去降补”任务落地见效。关停落后产能企业14家，淘汰小矿山7家，关破重组一批“僵尸企业”，成功化解PCB产业链债务风险。通过放开落户限制、推行货币化安置、租售并举等房地产调控举措，盘活商业地产47万平方米；减少商品住房库存42万平方米，去化周期由上年底18个月下降到13个月。有效防控金融风险，化解企业不良资产43亿元，规上企业资产负债率降低1.33个百分点；争取置换债券化解政府债务49.7亿元，政府平台融资成本控制在6.5%以内；银行不良贷款余额和不良贷款率实现“双下降”。多措并举降低企业成本35亿元，用电大户全部实现直供电。

　　认真落实中央和省系列稳增长政策，推动经济平稳健康较快发展。深化“暖企行动”，选派109名干部入驻企业，帮助解决问题360多个。设立过桥资金3.77亿元，充实国有担保机构资本金3.25亿元，“政银担”在保余额位居全省第三；金融机构贷款余额突破千亿大关。组建运营天使投资、风险投资、产业投资多支基金。投入产业扶持资金3.3亿元。推动企业完成直接融资120亿元，是上年2.8倍；新增“新三板”挂牌企业2家。全年新增规上企业45家、“专精特新”企业14家。

　　第二，坚持创新发展，加快“调转促”步伐。研究出台了加快创新驱动发展的实施意见。知识产权产出指标爆发式增长，新增发明专利510件，是上年2倍；新增著名商标22个，增长15.8%。企业技术创新平台不断发展，新增省级以上研发机构10家，新认定国家高新技术企业41家；PCB国家质检中心获批建设。新引进高层次科技人才团队11个，为历年之最。

　　全面厚植发展新动能。创新创业成效显著，建成众创空间17家，其中3家成为全国首批农业创新创业平台；新建一批电商产业园区，新业态快速发展，电子商务交易额增长35%，快递业务量增长105.6%；全年新登记市场主体增长20.6%。新兴产业集聚基地健康发展，铜基新材料集聚基地投资增长25%，产值达686亿元；首批4家市级基地建设全面启动。传统产业技术改造持续发力，全年技改投资突破400亿元，占工业投资比重70%，135家企业通过“两化融合”评估审核，传统动能焕发生机。服务业发展提质提效，新增4A级景区2家、省级服务业集聚区1个。新增农民专业合作社92家、家庭农场281个，规模以上农产品加工产值突破190亿元，是农业产值2.3倍，现代农业加快成长。

　　第三，强化项目带动，持续扩大有效投入。落实“四督四保”制度，开展“项目建设协调月”活动，促进各类项目建设提速增效。全年亿元以上项目完成投资570亿元，增长58.3%。民间投资累计完成886亿元，增长9%，高于全国、全省平均水平。安博电路板，松宝、耐科、零点机器人等106个亿元以上项目建成投产，皖能第二台百万千瓦机组并网发电，艾默生电器、诚创手机、沃特玛电池项目当年开工、当年竣工投产；有色“奥炉”改造、泰新新能源汽车等重大项目顺利推进。加大项目转化力度，南夹江综合治理项目前期工作取得突破。新谋划亿元以上项目315个，其中78个项目挤进省计划盘子，G3长江公铁大桥、庐铜铁路连接线等项目进入预可研阶段。加大征收拆违力度，累计征收房屋150万平方米、土地1.4万亩，拆除违章建设13.6万平方米，为项目建设提供了有力保障。抢抓国务院大督查表扬激励政策机遇，获批发行企业债251亿元，发债额是上年2.25倍；争取上级转移支付资金58.5亿元、国家专项建设基金29.6亿元。

　　第四，深化改革开放，不断增强发展活力。蹄疾步稳推进各项改革，年度41项任务全面完成。“放管服”改革持续深化，政府权力清单在全省率先上线，公共服务和中介服务清单制度建设走在全省前列，“五证合一”营业执照全省首发。“多规合一”联合审批系统在全省率先运行，建设项目总审批时限由257个工作日缩短到56个，试点经验全省推广。市、县（区）统一规范的公共资源交易平台基本建成。国企国资改革深化，铜陵有色员工持股计划顺利实施，铜化集团混合所有制改革进入股权转让阶段；盘活国有资产取得阶段性成效，清理盘活资产4.8亿元。PPP合作模式取得突破，城市排水一体化项目实现运营，华山大道地下综合管廊工程全线开工。“营改增”顺利实施。社会事业改革全面推进，义务教育“阳光分班”成为中央改革办推介的典型案例；校长职级制改革获全国教育改革创新特别奖。医药卫生体制综合改革取得新进展，医联（共）体实现四级全覆盖，“医养结合”试点有序开展。农村土地承包确权颁证试点任务全面完成，农村产权抵押融资突破亿元大关。国有林场和供销社改革稳步推进。不动产登记实现统一发证。

　　全方位扩大开放合作。招商引资势头良好，新引进亿元以上项目155个、10亿元以上项目12个，其中新兴产业项目占79.4%。在建省外投资亿元以上项目到位资金480亿元，增长9.6%，增幅居全省第6位；新批外资企业9家，实际利用外商直接投资2.52亿美元，增长8.7%。港口经济发展迅速，铜陵港跻身亿吨大港，与宁波舟山港建立了战略合作关系。进境水果指定口岸主体工程完工。港航投资集团组建运营。全市73家企业与“一带一路”49个国家和地区开展经贸合作，外贸进出口完成省调度计划。

　　第五，完善城市功能，提升城乡建设品质。对接国家和省区域发展战略，出台实施十三五规划纲要，编制完成新一轮城市总体规划、枞阳县城总体规划，江北港新区等重点专项规划，规划体系不断完善。交通建设再掀热潮，坝白路、沿新路、站前路建成通车，国道236铜芜段完工，农村公路畅通工程完成632公里。新区建设成效明显，市一中西湖校区、恒大小学开学，溪潭幼儿园建成，市儿童医院主体完工，优质教育、医疗资源快速向新区布局。城市功能不断完善，新建改造义安大道、天山大道等城市主次道路与微循环道路25条；新增清洁能源公交车79辆；新建立体停车场3座；整治天桥新村、爱国东村等老旧小区10个；大铜官山公园启动建设，滨江生态生活岸线向北延伸，板栗山儿童公园焕然一新；市烈士纪念馆、老年学习活动中心建成开放，市档案馆、史志馆、城建档案馆主体完工。乡村面貌深刻变化。16个乡镇政府驻地建成区完成整治，建成美丽乡村30个省级中心村、18个市级中心村，6679个自然村村容村貌得到改善，乡村魅力日益彰显。

　　第六，强化生态建设，提升绿色发展水平。坚持以项目为抓手，着力推进气水土污染治理。安排环境治理项目118个，总投资152.9亿元，已完成投资74亿元。皖能5号机组、国电1号机组超低排放完成改造，六国化工、金隆铜业、冬瓜山铜矿等一批废水治理工程建成使用。坚持开展大气污染防治攻坚行动，突出企业物料堆场、机动车尾气和秸秆焚烧整治，78个物料堆场实现“车间化”管理，淘汰黄标车和老旧车4270辆。全年PM10平均浓度77.8微克／立方米，下降12%，G20峰会期间空气质量保障任务圆满完成。坚持大力治理水体，在全省率先建立“河长制”，形成了行政首长负责的“八大水系”管理体系。黑砂河、秀水河、幸福渠基本实现水清岸绿。三大入江口国家考核断面水质全部达标。环境监管实现随机化、常态化。坚持推进生态修复，人工造林2.86万亩，绿化提升308万平方米，新增森林长廊示范段和城市绿道128.4公里，新增街头绿地（游园）20个，恢复矿山植被2077亩。山水园林城市品质和美誉度不断提升。

　　第七，实施精准扶贫，持续发力改善民生。脱贫攻坚首战告捷，1.9万贫困人口达到脱贫标准、21个贫困村满足出列条件。完善了精准扶贫脱贫政策体系，建立了“一结双包”定点帮扶机制，在全省率先实现“领导联系、单位包村、干部包户、工作队驻村”四个全覆盖。扶贫责任落实精准，核查识别精准，信息采集精准，因户施策精准，政策兑现精准。财政与社会各方投入资金4.4亿元，22个村集体和2350户光伏电站并网发电。精心实施38项民生工程，民生类财政支出128亿元，占总支出的85%，连续四年保持全省先进位次。棚改攻坚成效明显，货币化安置率65%，7328户棚户区居民圆上安居梦，联盟十一队等重点区域棚改取得突破，三年棚改任务“大头落地”。回迁安置居民1.32万户，历史遗留安置问题基本消化。实施农村电网升级改造项目160个，惠及109个行政村、1.26万户居民。改造农村危房3110户，1.2万农村人口安全饮水问题得到解决。就业和社会保障水平不断提升。城镇新增就业2.61万人，登记失业率控制在3.1%以内。城乡居民医保实现商业保险机构经办，大病医疗保险报销比例提高8.2%，机关事业单位养老保险制度实施，离退休人员基本养老金人均增长7.6%，城乡低保标准提高8%。人民群众的获得感不断增多。

　　第八，加强社会建设，促进社会和谐稳定。各级各类教育协调发展，公办幼儿园比例提高17.2个百分点；75所薄弱中小学校完成改造，枞阳县义务教育均衡发展通过省级验收；特教学校迁入新址，青少年综合实践基地投入使用；职业教育校企融合不断深化。群众性文化活动广泛开展，成功举办了中国图书馆年会和第十三届中国（铜陵）青铜文化博览会。全面落实两孩政策，出生人口政策符合率高于省控目标。全民健身运动蓬勃开展。城市管理体制进一步理顺，社区网格化管理实现全覆盖。省食品药品安全城市争创成功。安全生产形势保持稳定，事故起数下降15%。法治宣传教育获得全国先进。依法信访有效落实，社会治安稳定向好。社会科学、新闻出版、广播影视、统计、外宣、气象、地震、人防、消防、兵役等工作进一步加强，外事侨务、对台、港澳、民族宗教、妇女儿童、残疾人、红十字等事业取得新成绩。

　　一年来，我们切实加强法治政府建设。扎实开展“两学一做”学习教育和“讲看齐、见行动”学习讨论，深入践行“三严三实”，不断提高施政能力和服务水平。深入推进依法行政，落实政府学法制度，加强政府立法，规范政府规章制定程序，大力推进禁止燃放经营烟花爆竹地方法规的落实。健全依法决策机制，重大事项合法性审查实现全覆盖。自觉接受人大法律监督、工作监督和政协民主监督，办结人大代表建议171件、政协提案267件。树牢过“紧日子”的思想，严格执行中央八项规定和省、市委相关规定，“三公”经费下降30%。落实全面从严治党主体责任，加强党风廉政建设，认真抓好中央、省委巡视反馈问题的整改。深入开展酒桌办公、违规经商办企业和“四风”突出问题回头看等专项整治，严肃查处发生在群众身边的不正之风和腐败问题。强化行政监察和审计监督，加大正风肃纪力度，建立健全一批规章制度和长效机制。

　　各位代表！

　　一年来，我们取得的成绩来之不易，必须倍加珍惜。这是省委、省政府和中共铜陵市委坚强领导的结果，是全市人民锐意进取、顽强拼搏的结果。在此，我代表市人民政府，向全市人民，向各民主党派、各人民团体和各界人士，向驻铜解放军指战员、武警官兵和政法公安干警，向关心、支持铜陵改革发展的省直各部门、兄弟城市和海内外友好人士，表示衷心的感谢！

　　在肯定成绩的同时，我们也清醒地认识到发展中的问题、前进中的困难。宏观形势依然错综复杂，世界经济复杂性、不稳定性、不确定性进一步凸显；国内经济发展面临多重困难和挑战，存在重大结构性失衡，经济下行压力仍然持续。从我市情况看，经济结构性矛盾突出，传统产业比重过大，新经济、新产业、新业态尚处于培育成长期，接续替代产业项目数量不多、块头不大，产业转型升级处于爬坡过坎的关键阶段。项目储备不足，谋划不深；发展平台支撑力、吸引力不平衡，部分园区缺乏批量承接项目条件；创新驱动能力不足，领军人才和科技团队数量不多，高端龙头企业引进、收购、合作的力度有待加强；国家金融债务政策调整给融资工作带来新的挑战。江南江北发展不平衡、不协调问题突出，生态治理、环境改善任重道远，脱贫攻坚、补齐短板任务艰巨。少数政府部门和单位管理薄弱、能力不足，极少数干部不愿作为、不敢担当，“四风”问题和消极腐败现象仍然存在。我们必须高度重视，未雨绸缪，主动有效应对，认真加以解决。

　　各位代表！

　　铜陵建市已经走过一个甲子。60年的发展历程表明：实干推动崛起，奋斗创造辉煌！今天，我们手握接力棒，我们义无反顾，责无旁贷，走好新的长征路。我们坚信，只要全市上下坚持干字当头、干在实处，不忘初心、继续前进，我们就一定能够实现“五大发展”闯新路，铸就幸福美丽新铜陵！

　　二、2017年工作安排

　　今年是实施“十三五”规划的重要一年，是本届政府的收官之年，做好今年各项工作意义重大。当前，新一轮产业革命孕育突破，国家宏观政策特别是财政政策更加积极有效，中央加大对资源枯竭型城市的政策支持力度，这些都为我们加速转型发展提供了重要契机。我们要顺势而为、乘势而上，奋发有为、创优争先，全力把铜陵发展推向更高水平。

　　今年政府工作的总体要求是：全面贯彻党的十八大和十八届历次全会精神，深入贯彻习近平总书记系列重要讲话特别是视察安徽重要讲话精神，认真落实省、市第十次党代会和市委十届二次全会部署要求，统筹推进“五位一体”总体布局，协调推进“四个全面”战略布局，坚持稳中求进工作总基调，坚定践行新发展理念，适应把握引领经济发展新常态，以提高发展质量和效益为中心，以推进供给侧结构性改革为主线，以实施五大发展行动计划为总抓手，坚持目标导向、问题导向、创新导向、项目导向，统筹做好稳增长、促改革、调结构、惠民生、防风险各项工作，推动经济社会持续平稳健康发展，以优异成绩迎接党的十九大胜利召开！

　　今年经济社会发展的主要预期目标是：全市生产总值增长8.5%以上；财政收入增长5%以上；固定资产投资增长11%以上；社会消费品零售总额增长10%以上；城镇、农村居民人均可支配收入分别增长8.5%和9%以上；居民消费价格涨幅3%以下；城镇新增就业2.5万人、登记失业率控制在3.5%以内；实现22个贫困村出列、3.1万人脱贫；完成省政府下达的节能减排年度目标任务。

　　新的一年，我们要全力争创国家资源型城市产业转型升级示范区，成功创建天井湖国家旅游度假区，确保进入国家森林城市行列，奋力再捧“长安杯”！

　　重点要抓好五个方面工作：

　　（一）实施创新发展行动，打造转型升级新引擎

　　深入推进供给侧结构性改革。围绕满足需求，提高供给质量，扩大有效供给，深入推进“去降补”。坚定不移去产能，提升建材、钢铁、能源、纺织服装产业市场适应性，完善落后和过剩产能退出机制，分类处置“僵尸企业”。深化国资国企改革，完成铜化集团混合所有制改革，支持铜陵有色整体上市，国有独资企业上缴净利润提高到25%。综合施策去库存，引导新市民住房消费，推行以货币化为主的征迁安置方式，严控非住宅商品房土地供应，加强住房市场监管，促进房地产市场平稳健康发展。优化融资结构去杠杆，把降低企业杠杆率作为重中之重，建立政策性融资担保风险补偿机制，引导商业银行对市场前景好、暂时有困难的企业不断贷、不抽贷，支持企业市场化、法治化债转股。加大股权融资力度，完成1户企业IPO报会在审，新增“新三板”挂牌企业6家，实现直接融资100亿元。加强政府债务管控，平台融资成本控制在6.5%以下。加强金融生态建设，确保不发生系统性、区域性金融风险。大刀阔斧降成本，加大减税降费力度，进一步在降低人工、用能、用地等要素成本上加力增效，实现综合成本降幅高于全省平均水平。

　　加快建立科技创新体系。坚持在创新中转型、在转型中发展。构建企业和服务平台、科技与金融融合、创新创业模式、政策和制度集成四大创新体系，争创国家创新型城市。实施创新主体强化工程，新增国家高新技术企业15家以上、科技“小巨人”企业10家。实施创新平台升级工程，大力推进狮子山国家高新区建设，加强皖江中心、合工大铜陵工程技术研究院能力建设，支持企业申报国家级工程技术中心和重点实验室，建成PCB国家质检中心；新增省级以上研发平台6个，规上工业企业研发机构覆盖率达30%；新增省级以上众创空间2个、科技孵化器2万平方米。实施创新人才集聚工程，引进10个以上高层次创新团队。实施创新环境优化工程，整合优化创新激励政策，设立科技型企业贷款风险补偿资金池，开展创新创业券试点，支持天使投资、风险投资、产业投资基金做大规模，完善科技管理服务、产学研合作和科技评价机制，促进科技金融融合，不断激发创新活力。

　　大力振兴实体经济。工业强、铜陵强；实体兴、铜陵兴。要下大力气扩大产业投资，着力防止“脱实向虚”，引导更多要素资源投入工业、制造业领域，全力促进实体经济增数量、上规模、提效益，增强综合竞争力。深化“暖企行动”，完善精准帮扶机制，落细落实帮扶政策。深入推进“千百十亿”工程。强化新一轮技改升级行动，实施亿元以上技改项目50个，加快传统企业信息化、智能化改造。力推“个转企、小升规、规改股、股上市”，新进规上企业45家以上，促进中小企业“专精特新”发展，培育一批行业领军企业。开展“增品种、提品质、创品牌”专项行动，支持企业参与国际标准、国家标准、行业标准建设，引导企业弘扬“工匠精神”，新增省级新产品、工业精品15个以上。大力培养优秀企业家群体，营造有利于企业家成长的良好环境。

　　千方百计扩大有效投入。项目是发展的生命线，是“调转促”的主抓手，是稳增长的硬支撑。要坚持政府工作项目化，建立完善围绕项目转、盯着项目干、常态抓项目的工作机制，持续督查推进新建项目、续建项目、竣工项目和储备项目，保证开工率、竣工率、达产率和转化率。紧紧围绕产业发展、重大基础设施、城乡建设、民生和社会事业，加强项目谋划，建立政府性投资项目生成评估机制，努力形成大项目“顶天立地”、小项目“铺天盖地”的良好态势，推动形成行行上项目、人人抓项目、乡乡有项目的发展格局。全年安排亿元以上重点项目500个，新开工120个以上，竣工100个以上，完成投资550亿元。力争有色“奥炉”改造联动试车，泰新新能源汽车全线投产，争取“无中生有”引进一批重大项目落地建设。

　　开展产业集聚基地推进年活动。“三重一创”建设是促进“调转促”的战略举措。要健全推进机制，强化政策引领，完善考核办法，促进重大产业基地健康发展。省级铜基新材料产业集聚基地完成产值800亿元、税收10亿元，在全省争先进位。加快新能源汽车、电子信息、先进装备制造、精细化工4个市级集聚基地建设，力争1－2个升为省级基地。加快推进工业机器人、集成电路等5个重大新兴产业工程。加快实施超级电容、纳米复合材料等5个重大新兴产业专项，着力构建现代产业体系。

　　开展特色小镇培育建设年活动。特色小镇是孕育新业态、新经济、新产业、新模式的重要平台，是推动创新创业、产业转型、新型城镇化的重要载体。要突出产业培育，发挥龙头企业带动作用，充分运用“互联网＋”等手段，重点打造皖江创新小镇、大通文化旅游小镇、西湖文体小镇、天井小镇。鼓励各县区因地制宜，培育建设一批产业上“特而强”、机制上“新而活”的特色小镇，为转型发展增添新动能。

　　（二）实施协调发展行动，构建城乡一体新格局

　　开展协调发展提升年活动。协调发展是新铜陵发展的现实迫切需要。要围绕强基础、促均等、惠民生，统筹布局，细化安排，加大补短板力度。向江北地区交通、水利、电力设施短板发力，向教育、医疗、公共文化等社会事业短板发力，向社会保障、扶贫开发、饮水安全、乡村环境等民生短板发力，促进江南与江北、城市与农村、经济与社会加快协调发展步伐。

　　着力提振县区园区经济。深入实施县区经济振兴工程、园区经济倍增计划，促进区域经济协调发展。突出项目建设、实体企业、支撑平台、环境优化四大工作重点，聚焦投入、产出和贡献三大核心指标，加大调度通报、动态评价和考核激励力度，推动县区园区经济错位发展、集聚发展，在全市增比重，在全省进位次。支持县区建设产业集群镇，争创经济发达镇。支持市经开区争创国家产城融合示范区。强力推进铜官区国家服务业综合改革试点区建设，支持枞阳县国家全域旅游示范区建设，鼓励弘桥智谷等电商产业园做大做强，打造一批省级现代服务业集聚区，促进现代物流、金融和后台服务业加快发展。新增限上企业30家以上。

　　推进农业供给侧结构性改革。以促进农民增收为核心，把增加绿色优质农产品供给放在突出位置，加快建设现代农业生产体系、产业体系和经营体系。严守耕地红线，新增高标准农田5.8万亩，全年粮食总产达80万吨，实现无公害、绿色、有机农产品认证率增长10%。大力推进农产品加工区建设，规模以上农产品加工产值达200亿元；建成标准化鱼塘2000亩、蔬菜基地1500亩；因地制宜，做精牡丹、白姜、白茶等特色产业，促进农村一二三产业融合发展。培育新型农业经营主体，新增一批示范家庭农场和农业产业化联合体，推动普济圩大型农工商产业集团建设取得实质性进展。

　　提升交通通达能力。开辟对外通道。深入谋划推进连南接北、承东启西的铁路、高速、港口和航空项目，新建德上高速枞阳段，着力推进南北过江隧道和北沿江高速、高铁前期工作。畅通市域交通。全面启动G3长江公铁大桥建设，开工建设庐铜铁路连接线。快补交通短板。实现国道347、236和省道335路基全线贯通，推进国道237与省道425、221建设。农村公路畅通工程实现“三年计划两年完成”。

　　加大水利治理力度。全面启动建设总投资20亿元以上的水利工程。开工建设南夹江综合治理工程和胥坝跨江大桥。落实引江济淮工程建设任务。启动顺安河三级航道整治。实施中小河流治理项目6个，水库除险加固19座，改扩建沙池等城乡排涝泵站6座。玉带河整治工程，铜芜路泵站、青山圩泵站、北埂分洪闸项目和水毁修复项目确保汛前全面完成，显著提升城乡防洪排涝能力。

　　统筹推进城乡建设。实施新一轮城市总体规划，贯彻海绵城市建设理念，组织开展城市修补、生态修复，提升城市功能和品质。畅通城市路网，开工建设翠湖二路东延等一批骨干路网，打通一批微循环道路和断头路。治理违法建设、违法用地，继续改造一批老旧小区，完成城区棚户区改造扫尾工程，建设特色风貌街区，促进人居环境不断改善。优先发展公共交通，再投放清洁能源公交车90辆，启动新一轮出租车更新，建成一批立体停车场和充电桩。推进智慧城市建设，实施社区光纤改造“最后一公里”工程，提高宽带普及率和免费无线网络覆盖率，完成“宽带中国”示范城市创建目标。加快东部城区建设步伐。推进美丽乡村扩面提质，建设省级中心村30个、市级中心村15个，完成15个乡镇政府驻地建成区整治，加强农村自然村环境治理，建设人富庄美家园，打造乡村魅力板块。

　　（三）实施绿色发展行动，建设生态文明新样板

　　打造循环经济升级版。坚持减量化、资源化、再利用，精心组织实施“8311”行动年度任务，安排重点项目100个。推动循环经济由市域资源利用小循环向国际国内资源利用大循环延伸，由主要依靠产业循环拉动向依靠产业、社会、城市循环协同拉动拓展，由主要促进经济可持续发展向促进经济、社会、人口、资源、环境全面协调可持续发展升级，形成“循环引领、产城融合、三生共赢”的发展模式。争取市经开区循环化改造通过国家验收，巩固国家循环经济示范市创建成果，保持循环经济全国领跑地位。深入推进国家工业绿色转型发展试点市建设。创建全国国土资源节约集约模范市。

　　持续强力治理环境污染。实施“蓝天、碧水、净土”工程，打好气水土污染防治“三大战役”。大力整治大气污染，建立倒逼机制，推动企业上项目、促治理。全面完成企业物料堆场“车间化”管理改造，大力推动秸秆综合利用，加大黄标车和老旧车淘汰力度，关闭一批小矿山，实现PM10、PM2.5平均浓度达到省控目标。强化水污染综合防治，落实“河长制”三年行动计划，加强顺安河、红星河、惠溪河治理；实现工业园区污水集中处理设施全覆盖，地表水环境质量达到三类以上标准。开展土壤环境质量调查，推进土壤污染治理与修复试点工作。加强农业面源污染防治，争创国家农业可持续发展试验区。强化各级环保责任落实，严格执法，严肃追责问责，以“零容忍”的态度铁腕治污。

　　加强生态保护与修复。开展林业增绿增效行动，实施人工造林7083亩，新增和改造提升绿化185万平方米，抚育森林16.8万亩。建成杨村公园，推进大铜官山公园建设，动工兴建鹞山公园，新增城市绿道40公里、街头绿地（游园）16个。巩固扩大“三线三边三小”治理成果，推进矿山裸露山体整治，加强地质灾害防治。建设西湖湿地二期工程，开展东湖湿地保护工作，进一步彰显铜陵湿地特色。

　　（四）实施开放发展行动，增强区域竞争新优势

　　加强开放合作平台建设。加快B型保税物流中心申报建设进程。抢抓五金市场回暖契机，推动“圈区管理”园区加快发展。建成运营进境水果、粮食指定口岸。开展江北港新区深度设计，推动横港、永丰万吨级码头建设，提升港口吞吐能力。发挥港航投资集团龙头作用，整合港口岸线资源，深化与宁波舟山港的战略合作，积极发展多式联运、江海联运。深化口岸一次申报、一次查验、一次放行和关检“一站式作业”，加强跨地区通关通检协作，提高通关便利化水平。

　　继续加大招商引资力度。坚持招大引强、招新引优、招才引智，保持招商引资强劲势头。抓住国家允许地方政府在法定权限范围内制定招商引资优惠政策的契机，深入研究、用活用好。重点引进国内外中高端产业及龙头企业，加强与世界500强、央企和知名民企合作，鼓励有条件的企业收购、兼并、合作国际国内企业和研发机构。全年新引进亿元以上项目135个，各县区、园区引进超10亿元产业项目2个以上，亿元以上省外项目实际到位资金增长10%以上，外商直接投资增长8.7%。

　　高水平推进对外合作。围绕打造长三角城市群西翼新兴增长极，加速“东融北联”，制定落实长三角城市群发展规划实施方案，借鉴上海自贸区改革试点经验，推动体制机制等高对接苏浙沪，积极承接加工贸易转移；着力推动合铜发展带建设，促进与合肥都市圈联动发展。完善外贸进出口机制和政策措施，促进外贸稳定增长。支持企业加强与“一带一路”沿线国家和地区经贸合作，加快实施铜陵有色境外项目。

　　深化重点领域改革。强力推进“放管服”改革，认真落实权责清单和服务清单，实现“四单”网上运行，以权力的“减法”、服务的“加法”，换取市场活力的“乘法”。

　　全面推行“多规合一”，实现县区全覆盖。深入推进投融资体制改革，创新融资方式，对有稳定回报的基础设施、公共服务等项目，积极运用PPP、资产证券化等方式，引导金融机构、国有企业、民营企业进行投资。拓展融资渠道，加强政银企合作，运用多种金融产品，加快政府投融资平台转型步伐。着力深化农村改革，实施农村土地所有权、承包权、经营权分置改革，开展资源变资产、资金变股金、农民变股东“三变”试点，推动农村产权抵押融资试点扩面。

　　（五）实施共享发展行动，增进人民群众新福祉

　　织密扎牢民生保障网。坚持以人民为中心的发展思想，做好普惠性、兜底性、基础性民生建设，持续加大民生投入，继续实施38项民生工程，提升建设管养、绩效管理水平。组织实施“七大群体”激励计划，让人民群众在共建共享发展中有更多获得感。

　　全面完成脱贫攻坚年度任务。坚持精准扶贫、精准脱贫，全面对标“六看六确保”，健全“四项机制”，确保完成年度目标任务。健全精准扶贫机制，严把精准施策关，深入实施脱贫攻坚“十大工程”，一户一策量身定制。健全监测评估机制，严把精准识别关，加强数据库建设，实行脱贫对象动态管理；严把精准脱贫关，严格执行扶贫对象退出标准和机制，做到真扶贫、扶真贫、真脱贫。健全防范返贫机制，实行返贫预警，强化返贫信息管理，加强定期跟踪回访，及时将返贫户纳入帮扶范围。健全责任落实机制，严把精准帮扶关，强化各级各部门责任，稳定“城乡结对、单位包村、干部包户”和驻村工作队帮扶办法，深入开展“百企帮百村”行动，形成全社会共同参与扶贫的良好局面。

　　加强就业和社会保障工作。就业是民生之基。坚持以创业带动就业，重点做好高校毕业生、农民工、退伍军人和就业困难人员就业工作，确保零就业家庭动态“清零”。推进“全民参保计划”，做好工伤保险省级统筹。提高城乡居民养老保险、城乡低保和五保供养标准。推动住房公积金制度向进城农民工覆盖。完善社会救助体系，规范实施重特大疾病医疗救助，健全基层“救急难”工作机制。

　　办好人民满意的教育。实施第三期学前教育三年行动计划，新增一批省级和市级一类幼儿园。改善薄弱学校办学条件，确保枞阳县义务教育均衡发展通过国家认定。促进优质教育资源向新区和农村学校覆盖，不断满足市民上好学校的愿望。全面提升教师队伍素质，培养名校名师，推进教育领域综合改革，提高教育质量。深化职业教育产教融合，支持铜陵职业技术学院、安工学院和技师学院发展，培育一批高技能工匠人才，建设技工大市。支持铜陵学院加快建设地方应用型高水平大学。办好特殊教育，支持、规范民办教育发展。

　　高水平创建国家公共文化服务体系示范区。以“领先中部、看齐东部、示范全国”为标杆，推进图书馆、全民阅读、文化馆、全民艺术普及四大体系建设，形成一批可复制、可推广的经验，确保高标准通过国家评估。建成市非遗展示馆，加快区级、村级文化活动场所达标建设。冠名打造“中国铜博物馆”，建设国家方志馆长江分馆。切实整合各部门资源，推进全民科学素质行动计划纲要的实施，努力提升市民科学文化素质。

　　着力建设健康铜陵。制定实施健康铜陵2030规划纲要，争创国家健康城市。深化医药卫生体制综合改革，突出“三医联动”，以利益为纽带，健全医疗服务联合体，推进分级诊疗，全面推行家庭医生签约式服务。推进居家和社区养老服务改革试点，扩大“医养结合”试点成果，争创省级医养结合示范区。深入开展爱国卫生运动，创建健康社区、健康村镇、健康单位、健康家庭。构建四级全民健身设施网络，完成市体育中心体育场后续工程，办好市十二届运动会暨第四届全民健身运动会。落实全面两孩政策，提高优生优育水平。加强食品药品安全监管，保障群众饮食用药安全。加大农村水厂整合力度，提高饮水质量，保障饮水安全。

　　加强和创新社会治理。深入践行社会主义核心价值观，拉升文明城市标杆，打造志愿服务品牌，提升城市文明内涵，通过全国文明城市复审验收。认真组织实施燃放经营烟花爆竹管理规定，做到禁放工作法制化、常态化。加强互联网安全管理，构建和谐文明网络空间。增强全民国防意识，争创省双拥模范城“八连冠”。协调推进民族宗教、妇女儿童、老龄、残疾人等事业健康发展。深入推进“七五”普法。高度重视人民群众来信来访，加大矛盾纠纷排查化解力度。认真贯彻中央关于安全生产领域改革发展的新要求，扎实开展“铸安”行动，坚决遏制较大以上安全事故，防范重大安全风险，促进安全生产形势持续平稳。健全应急管理体系，增强防灾减灾能力。深化“平安铜陵”建设，扎实开展“守护平安”系列行动，完善社会治安防控体系，加强反恐防暴和禁毒工作，保持社会大局和谐稳定。

　　三、全面加强政府自身建设

　　形势催人奋进，发展任务艰巨，政府肩负重责，必须把自身建设放在更加突出的位置。我们要贯彻新理念，确立新标杆，认真践行忠诚干净担当要求，着力打造为民务实清廉政府，增强执行力，展现新作为。

　　强化政治定力，始终做到忠诚看齐。牢固树立“四个意识”，特别是核心意识、看齐意识，坚定不移地向党中央看齐，向党的理论和路线方针政策看齐，向党中央决策部署看齐，始终在思想上政治上行动上自觉与党中央保持高度一致，结合实际创造性转化，确保中央各项决策部署精准落实。坚定理想信念，强化理论武装，增强中国特色社会主义道路自信、理论自信、制度自信、文化自信，内化于心、外化于行，化为推动发展、造福人民的强大动力。

　　强化法治思维，推进法治政府建设。推动政府系统工作人员自觉尊法学法守法用法，严格遵循法定权限和程序履行职责，增强运用法治方式解决问题、化解矛盾的能力。加强政府法治机构建设，完善政府立法机制，提高立法质量。严格依法决策，落实重大决策法定程序，加强合法性审查，健全决策咨询制度，提高决策水平。突出加强和改进事中事后监管，强化智能监管、信用监管，推进城市综合执法改革。认真落实行政执法责任制，严格执法监督，规范执法行为。自觉接受人大监督、政协监督、司法监督，主动接受社会监督和舆论监督，认真办理人大代表建议和政协提案，广泛听取民主党派、工商联、无党派人士和各人民团体意见。全面推进政府信息公开，落实新闻发布制度，确保权力在阳光下运行。

　　强化服务创新，提升政务服务水平。适应快速变化的新形势，深入学习党中央治国理政新理念新思想新战略，及时补充新技术、新业态、新模式、新经济知识，提升专业素养、专业水准，增强服务本领。认真贯彻党的群众路线，多渠道了解社情民意，抓住“痛点”和“阻点”，深入基层，解剖麻雀。创新政府服务方式，扩大购买公共服务，加强资金使用绩效评价，提高公共服务质量。推进“互联网＋政务服务”，开展“减政便民”专项行动，打造网上政府，提高服务效率。深化机关作风效能建设，推行包办、代办服务，构建“亲”“清”新型政商关系，不断优化投资环境。办好市长热线、市长信箱、政务微博等政民互动平台，切实为群众排忧解难。

　　强化责任担当，不断增强执行能力。各级领导干部既要统筹兼顾当好“指挥员”、又要冲锋陷阵当好“战斗员”，既要担当、又要压责，推动形成“领着干、推着干、比着干”的干事氛围。建立健全正向激励机制，完善政绩考核评价体系，对落实部署坚决有力的给予褒奖，对工作成效明显的给予奖励。建立容错纠错机制，严格落实“三个区分开来”的要求，旗帜鲜明地为敢担当的干部担当、为敢负责的干部负责。完善攻坚克难推进机制，事关全局的重点难点工作都要立下军令状，制定路线图，列出时间表，做到时间倒排、任务倒逼、责任倒追。健全督查问效机制，建立重大事项领导督导清单、重点事项督查清单制度，全程督导、跟踪督查，确保政府工作掷地有声、落地生根。

　　强化正风肃纪，加强党风廉政建设。严格落实全面从严治党主体责任，全面加强和规范党内政治生活，自觉加强和接受党内监督，提升政府系统从严管党治党水平。全面落实党风廉政建设责任制和“一岗双责”，加强行政监察，推进审计监督全覆盖，构建严密的权力运行制约和监督体系，全方位扎紧制度笼子。自觉践行“三严三实”，深入开展“讲看齐、见行动”学习讨论，巩固“两学一做”学习教育成果，严格执行中央八项规定和省、市委各项规定，严肃查处发生在群众身边的“四风”和腐败问题。坚决查办违纪违法案件，着力营造风清气正的政治生态。

　　各位代表！

　　担当者书写发展，奋进者赢得未来。让我们紧密团结在以习近平同志为核心的党中央周围，在省委、省政府和中共铜陵市委的坚强领导下，勠力同心、奋发有为，干在实处、走在前列，为加快建设幸福美丽新铜陵而努力奋斗！
